# A Manual of Ophthalmic Practice

**Published:** 1889-03

**Authors:** 


*A Manual of Ophthalmic Practice.* By CHARLES HIGGENS, F.R.C.S. London: H. K. LEWIS. 1888.

This small manual—one of Lewis's Practical Series—consists of 300 pages, and contains a digest of ophthalmic surgery, divided into three equal parts: 1st, Examination of the eye, optics and refraction; 2nd, The diseases of the eye and their treatment; 3rd, Operations. The author is very well qualified by his large experience to deal in a practical manner with the subject; and it is interesting to gather his views, especially as regards treatment. In the space allotted there is little room for pathology or for theory; and it is difficult to see how the book fills any want in ophthalmic literature.

It can scarcely be looked upon as a sufficient handbook for the practitioner of medicine, or as a satisfactory student's manual.

